# Maternal periconceptional consumption of sprouted potato and risks of neural tube defects and orofacial clefts

**DOI:** 10.1186/s12937-018-0420-4

**Published:** 2018-11-28

**Authors:** Wenli Ni, Tian Tian, Le Zhang, Zhiwen Li, Linlin Wang, Aiguo Ren

**Affiliations:** 1 Peking University Institute of Reproductive and Child Health, National Health Commission Key Laboratory of Reproductive Health, Beijing, 100191 China; 2 Department of Epidemiology and Biostatistics, School of Public Health, Peking University Health Science Center, Beijing, 100191 China

**Keywords:** Sprouted potato, Periconceptional period, Neural tube defects, Orofacial clefts

## Abstract

**Background:**

The association between maternal consumption of sprouted potato during periconceptional period on the development of neural tube defects (NTDs) or orofacial clefts (OFCs) remains unclear. We aimed to examine the association between maternal consumption of sprouted potatoes during periconceptional period and risks of NTDs or OFCs.

**Methods:**

Subjects included 622 NTD cases, 135 OFC cases and 858 nonmalformed controls, were recruited from a case-control study in Shanxi Province of northern China between 2002 and 2007. Information on demographics, maternal sprouted potato consumption, lifestyle behaviors and folic acid supplementation was collected.

**Results:**

Consumption of sprouted potatoes was associated with elevated odds of total NTDs (OR = 2.20; 95% CI, 1.12–4.32) and anencephaly (OR = 2.48; 95% CI, 1.10–5.58); no association for spina bifida or encephalocele. Sprouted potato consumption increased the risk of total OFCs (OR = 3.49; 95% CI, 1.29–9.49) and cleft lip with or without cleft palate (CL ± P) (OR = 4.03; 95% CI, 1.44–11.28).

**Conclusion:**

Maternal consumption of sprouted potatoes during periconceptional period may increase the risks of NTDs and OFCs. Given that potato is commonly consumed around the world, improper preservation and use should be a matter of concern in respect of the potential teratogenicity.

**Electronic supplementary material:**

The online version of this article (10.1186/s12937-018-0420-4) contains supplementary material, which is available to authorized users.

## Background

Neural tube defects (NTDs) and orofacial clefts (OFCs) are relatively common congenital anomalies that have a significant impact on infant mortality, health, and quality of life [1–3]. NTDs result from failure of neural tube to close by the 28th day of postconception [4]. OFCs derive from an embryopathy with the tissues of the lip or palate not joining properly [5]. The etiologies of NTDs and OFCs are generally thought to be multifactorial, involving both genetic and environmental factors. Environmental factors such as maternal unhealthy diet, and exposure to smoking and alcohol consumption contribute to these congenital anomalies [6–10]. Nutrient deficiency as a part of harmful dietary factors, especially folic acid deficiency, has been shown to be associated with increased risks of NTDs and OFCs [11–13]. Toxins in food are another important part of harmful dietary factors, however, the role of toxins in the occurrence of these two birth defects remains largely unknown.

Toxins in staple food have a great influence on human health because of the large amount of consumption. Today potato, as the 4th major food crop after rice, wheat and maize, is cultivated worldwide under various climatic conditions, and is consumed commonly throughout the world [14, 15]. However, in the process of storage and transportation, potato is easy to become green and spout in inappropriate environment [16, 17]. Sprouted potato contains high concentration of solanine that is a toxic glycoalkaloid [18]. The reproductive toxicity of solanine has been assessed in vitro or in vivo animal studies [15, 19–23]. Steroidal glycoalkaloid from potatoes could inhibit pre-implantation embryo development when exposing to oocytes and fertilized ova in vitro [15]. Additionally, solanine was found to have teratogenicity, such as inducing NTDs, OFCs, microcephaly, and severe muscular kinking in frog embryo, mice, or hamster [19–23]. However, only one study with a small sample size reported that sprouted potato consumption might be a risk factor for NTDs [24], while no study investigated the effect of maternal consumption of sprouted potato during periconceptional period on the development of OFCs in humans.

In the present study, we aimed to examine the association between maternal periconceptional consumption of sprouted potatoes and risks for NTDs and OFCs.

## Methods

### Study design

The data were from a case–control study of birth defects in four counties (Pingding, Xiyang, Taigu and Zezhou) of Shanxi province in northern China, which has been described elsewhere [25]. Concisely, a population-based surveillance system of birth defects was established in the study area in 2002. The surveillance system monitored major external structural birth defects through active case ascertainment. Cases with major external structural malformations were ascertained from live births, stillbirths, and elective terminations. County or township health workers verified the diagnoses by physical examination of the fetal body for all pregnancy outcomes and filled out a reporting form for each case. When a case infant was identified, a newborn control without any external structural birth defect was matched to the case infant by county, sex, maternal ethnic group, and date of the last menstrual period (±4 months). The study was approved by the Institutional Review Board of Peking University Health Science Centre. All participants provided written informed consent.

### Data collection

Trained local health care workers conducted face-to-face interviews with mothers of cases and controls within 1 week of delivery or termination of pregnancies to collect the exposure information using a structured questionnaire. Data were collected on the mother’s demographic characteristics, reproductive history, lifestyle, folic acid supplementation, smoking exposure (active or passive), and consumption of sprouted potato and other foods from 1 month before through 2 months after conception. Definition of maternal sprouted potato consumption is eating the sprouts of potato or any skin that started to turn green. Definition of passive smoking is staying in the tobacco smoking environmental for at least 30 min and at least 1 time per week. Definition of higher consumption of B vitamins is that maternal consumption of fresh vegetables and fresh fruit at ≥4 meals/week or maternal periconceptional use of folic acid, otherwise, it was lower consumption of B vitamins. Consumption of sprouted potato was classified as < 1 meal/week, 1–3 meals/week, 4–6 meals/week, or > 6 meals/week; we combined two frequency categories (“4–6 meals/week” and “> 6 meals/week”) into a single group (referred to as “≥ 4 meals/week”), because subjects with the highest consumption of sprouted potato frequency of “> 6 meals/week” were few.

### Study population

For this study, we used data collected from June 2002 through December 2007. The cases included newborns with NTDs or OFCs assigned an ICD-9 code of 740.0 (anencephaly), 741.0 (spina bifida), 742.0 (encephalocele), 749.0 (cleft palate), 749.1 (cleft lip), or 749.2 (cleft lip with cleft palate). A total of 1615 participants were available for analyses, including 622 NTD cases (281 with anencephaly, 293 with spina bifida, and 48 with encephalocele), 135 OFC cases (126 with cleft lip with or without cleft palate, 9 with cleft palate only), and 858 controls. Because OFCs and NTDs are rare diseases and few studies investigated the assocaition between sprouted potato consumption and birth defects, no information about the proportion of maternal sprouted potato exposure in control group or OR value can be referred for calculating sample size. In the present study, we used an empirical sample size. The statistical powers were 96.4% for NTDs and 99.7% for OFCs of this given the sample size (622 NTD cases, 135 OFC cases, and 858 controls) by PASS.11 software calculation, which were adequate to test the existence of significantly association between the sprouted potato consumption and NTDs and OFCs in this study.

### Statistical analyses

The original study used a pair-matching design, but for some data about sprouted potato consumption were not available for evaluation, the matching was broken in this analysis. The differences in demographic and lifestyle characteristics between the case and control group were evaluated by Chi-square test or Fisher’s exact test. Risk was estimated by the use of OR, and the precision of each OR was assessed by its 95% CI. We used the unconditional logistic regression and Chi-square trend test to evaluate the associations between sprouted potato consumption and risks of NTDs and OFCs, respectively. We calculated two adjusted OR (aOR) with different logistic models. The aOR_1_ was adjusted only for demographic and obstetrics variables, including maternal age, BMI, education, occupation, parity, history of pregnancy affected by birth defects, infant sex, folic acid supplementation, season of conception, alcohol drinking, and maternal smoking exposure. Considering the other dietary characteristics which may generate confounding bias, we also estimated aOR_2_ by additionally adjusting for consumption of meat or fish, consumption of egg or milk, consumption of fresh vegetable, consumption of fresh fruit, and consumption of legumes. Another, the associations between sprouted potato consumption and risks of NTDs and OFCs among mother with higher consumption of B vitamins consumption versus mother with lower consumption of B vitamins were conducted. We considered *p <* 0.05 statistically significant. All data were analyzed using SPSS 23.0 software (SPSS, Chicago, IL, USA).

## Results

### Characteristics of study population

The demographic and lifestyle characteristics of study participants are summarized in Table 1. More than 99% mothers were of Han ethnicity. Compared with control mothers, mothers of infants with NTDs were more likely to have a lower BMI and a lower education level, to be multiparous, to report a history of pregnancy affected by birth defects, to have alcohol drinking exposure, and to have smoking exposure; mothers of infants with OFCs were more likely to be not farmers, to have a lower education level, and to have a male infant.

**Table 1:** Characteristics of women who had pregnancies affected by NTDs or OFCs (cases) and women who delivered healthy infants (controls)

Characteristics	Controls (*n* = 858)	Cases			
		NTDs (*n* = 622)		OFCs (*n* = 135)	
	n(%)^a^	n(%)^a^	*p-*Value	n(%)^a^	*p-*Value
Maternal age (years)					
< 25	358(42.4)	249(40.8)	0.800	61(47.3)	0.140
25–29	268 (31.7)	196 (32.0)		45 (34.9)	
≥30	219 (25.9)	166 (27.2)		23 (17.8)	
BMI (kg/m^2^)					
< 18.5	53 (6.3)	45 (7.5)	0.002	11 (8.5)	0.339
18.5–23.9	417 (49.8)	350 (58.0)		70 (53.8)	
≥ 24	368 (43.9)	208 (34.5)		49 (37.7)	
Ethnicity					
Han	853 (99.6)	620 (99.8)	1.000	135 (100)	1.000
Other	3 (0.4)	1 (0.2)		0 (0)	
Occupation					
Farmer	568 (66.9)	420 (69.2)	0.356	73 (54.1)	0.004
Other	281 (33.1)	187 (30.8)		62 (45.9)	
Education					
Primary or lower	51 (6.0)	101 (16.3)	< 0.001	18 (13.5)	0.002
Junior high	656 (76.9)	445 (71.8)		87 (65.4)	
High school or above	146 (17.1)	74 (11.9)		28 (21.1)	
Parity					
Primiparas	538 (62.8)	345 (56.4)	0.013	95 (70.9)	0.069
Multiparas	319 (37.2)	267 (43.6)		39 (29.1)	
History of pregnancy affected by birth defects					
Yes	18 (2.1)	42 (6.8)	< 0.001	5 (3.7)	0.360
No	838 (97.9)	573 (93.2)		130 (96.3)	
Infant sex					
Male	404 (47.6)	268 (43.9)	0.153	84 (62.2)	0.002
Female	444 (52.4)	343 (56.1)		51 (37.8)	
Folic acid supplementation					
Yes	113 (13.7)	65 (10.7)	0.090	20 (14.4)	0.690
No	709 (86.3)	540 (89.3)		113 (85.6)	
Maternal smoking exposure					
Yes	491 (57.6)	423 (68.6)	< 0.001	84 (62.2)	0.315
No	361 (42.4)	194 (31.4)		51 (37.8)	
Alcohol drinking					
Yes	5 (0.6)	9 (1.4)	0.107	4 (3.0)	0.024
No	848 (99.4)	613 (98.4)		131 (13.4)	
Season of conception					
Spring	202 (24.1)	156 (25.4)	0.657	36 (26.9)	0.857
Summer	206 (24.6)	142 (23.2)		29 (21.6)	
Autumn	207 (24.7)	164 (26.8)		33 (24.6)	
Winter	222 (26.5)	151 (24.6)		36 (26.9)	

^a^Values for some variables may not equal the total number of cases or controls because of missing data

Additional file 1: Table S1 shows the dietary characteristics of study participants. Compared to control mothers, mothers of infants with NTDs were more likely to have lower frequencies of meat or fish consumption, egg or milk consumption, fresh vegetables consumption, fresh fruit consumption, and beans or bean products consumption. There was no significant difference between OFC cases and controls with respect to dietary characteristics.

Table 2 shows the association between sprouted potato consumption and risks of total NTDs and subtypes of NTDs. When maternal consumption of sprouted potato at < 1 meal/week was used as the reference, increases in the risks for total NTDs of 2.20-fold (95% CI, 1.12–4.32) were observed for maternal consumption at ≥4 meals/week, respectively. The positive relationship was also present for anencephaly but not for spina bifida or encephalocele; the adjusted ORs for anencephaly in association with maternal consumption of sprouted potato at ≥4 meals/week were 2.48 (95% CI, 1.10–5.58). Trend analysis showed a clear positive dose-response relationship between risks for NTD or anencephaly and sprouted potato consumption (*P* for trend < 0.05).

**Table 2:** Sprouted potato consumption and risks of total NTDs and NTD subtypes

Sprouted potato consumption	Controls (*n* = 858)	Total NTDs (*n* = 622)		Anencephaly (*n* = 281)		Spina bifida (*n* = 293)		Encephalocele (*n* = 48)	
	n(%)	n(%)	OR (95% CI)	n(%)	OR (95% CI)	n(%)	OR (95% CI)	n(%)	OR (95% CI)
Crude OR									
< 1 meal/week	737 (85.9)	512 (82.1)	1.00	229 (81.5)	1.00	243 (83.0)	1.00	38 (79.2)	1.00
1–3 meals/week	99 (11.5)	79 (12.7)	1.15 (0.84–1.58)	34 (12.1)	1.11 (0.73–1.68)	37 (12.6)	1.13 (0.75–1.69)	8 (16.7)	1.57 (0.71–3.46)
≥ 4 meals/week	22 (2.6)	33 (5.3)	2.16 (1.24–3.75)	18 (6.4)	2.63 (1.39–5.00)	13 (4.4)	1.79 (0.89–3.60)	2 (4.2)	1.76 (0.75–1.69)
Adjusted OR_1_^a^									
< 1 meal/week	737 (85.9)	512 (82.1)	1.00	229 (81.5)	1.00	243 (83.0)	1.00	38 (79.2)	1.00
1–3 meals/week	99 (11.5)	79 (12.7)	1.28 (0.88–1.87)	34 (12.1)	1.22 (0.74–2.02)	37 (12.6)	1.23 (0.77–1.97)	8 (16.7)	2.13 (0.86–5.27)
≥ 4 meals/week	22 (2.6)	33 (5.3)	2.22 (1.18–4.18)	18 (6.4)	2.42 (1.15–5.11)	13 (4.4)	2.03 (0.94–4.39)	2 (4.2)	2.22 (0.46–10.79)
Adjusted OR_2_^b^									
< 1 meal/week	737 (85.9)	512 (82.1)	1.00	229 (81.5)	1.00	243 (83.0)	1.00	38 (79.2)	1.00
1–3 meals/week	99 (11.5)	79 (12.7)	1.11 (0.75–1.63)	34 (12.1)	1.06 (0.63–1.78)	37 (12.6)	1.23 (0.76–2.00)	8 (16.7)	1.82 (0.70–4.73)
≥ 4 meals/week	22 (2.6)	33 (5.3)	2.20 (1.12–4.32)	18 (6.4)	2.48 (1.10–5.58)	13 (4.4)	2.17 (0.97–4.85)	2 (4.2)	3.18 (0.61–16.65)
*P* for trend		0.009		0.011		0.117		0.203	

^a^Adjusted for maternal age, education, BMI, occupation, infant sex, parity, history of pregnancy affected by birth defects, folic acid supplementation, season of conception, alcohol drinking, and maternal smoking exposure

^b^Adjusted for maternal age, education, BMI, occupation, infant sex, parity, history of pregnancy affected by birth defects, folic acid supplementation, season of conception, alcohol drinking, maternal smoking exposure, consumption of meat or fish, consumption of egg or milk, consumption of fresh vegetable, consumption of fresh fruit, and consumption of legumes

Table 3 details the association between sprouted potato consumption and risks of total OFCs and CL ± P. When maternal consumption of sprouted potato at < 1 meal/week was used as the reference, increases in the risks for total OFCs and CL ± P of 3.49-fold (95% CI, 1.29–9.49) and 4.03-fold (95% CI, 1.44–11.28) were observed for maternal consumption at ≥4 meals/week, respectively.

**Table 3:** Sprouted potato consumption and risks of total OFCs and OFC subtypes

Sprouted potato consumption	Controls (*n* = 858)	Total OFCs (*n* = 135)		CL ± P (*n* = 126)	
	n(%)	n(%)	OR(95% CI)	n(%)	OR(95% CI)
Crude OR					
< 1 meal/week	737 (85.9)	111 (82.2)	1.00	104 (82.5)	1.00
1–3 meals/week	99 (11.5)	16 (11.9)	1.07 (0.61–1.89)	14 (11.1)	1.00 (0.55–1.82)
≥ 4 meals/week	22 (2.6)	8 (5.9)	2.41 (1.05–5.56)	8 (6.3)	2.58 (1.12–5.94)
Adjusted OR_1_^a^					
< 1 meal/week	737 (85.9)	111 (82.2)	1.00	104 (82.5)	1.00
1–3 meals/week	99 (11.5)	16 (11.9)	1.37 (0.70–2.69)	14 (11.1)	1.34 (0.67–2.68)
≥ 4 meals/week	22 (2.6)	8 (5.9)	2.99 (1.12–8.03)	8 (6.3)	3.17 (1.18–8.55)
Adjusted OR_2_^b^					
< 1 meal/week	737 (85.9)	111 (82.2)	1.00	104 (82.5)	1.00
1–3 meals/week	99 (11.5)	16 (11.9)	1.10 (0.55–2.21)	14 (11.1)	1.20 (0.57–2.50)
≥ 4 meals/week	22 (2.6)	8 (5.9)	3.49 (1.29–9.49)	8 (6.3)	4.03 (1.44–11.28)
*P* for trend		0.094		0.099	

^a^Adjusted for maternal age, maternal education, BMI, occupation, infant sex, parity, history of pregnancy affected by birth defects, folic acid supplementation, season of conception, alcohol drinking, and maternal smoking exposure

^b^Adjusted for maternal age, maternal education, BMI, occupation, infant sex, parity, history of pregnancy affected by birth defects, folic acid supplementation, season of conception, alcohol drinking, maternal smoking exposure, consumption of meat or fish, consumption of egg or milk, consumption of fresh vegetable, consumption of fresh fruit, and consumption of legumes

We also assessed association between maternal sprouted potato consumption and risks of NTDs and OFCs in stratified by consumption of B vitamin (Additional file 1: Table S2 and S3).

Among mother with lower consumption of B vitamins, maternal consumption of sprouted potato at ≥4 meals/week, still were significantly associated with NTDs and OFCs, the aOR were 1.84 (1.02–7.86) for total NTD, 3.21(1.00–10.35) for anencephaly, 12.17(2.22–66.63) for total OFCs, and 15.65(2.68–91.40) for CL ± P. However, no significant associations were observed between maternal consumption of sprouted potato and NTDs and OFCs among mother with a higher consumption of B vitamins. Recall bias is the main concern in case-control studies. The women who have infant with birth defects might over-report unhealthy exposure. To assess whether such a recall bias existed in the present study, we further analyzed the distribution of consumption of sprouted potato during periconceptional period between women had infant with limb malformations (polydactylism, syndactylia, limb shortening, strephenopodia, or strephexopodia) without other malformations and controls (Additional file 1: Table S4). No difference was found in the distribution of sprouted potato consumption between cases and controls (*P* = 0.066).

### Sensitivity analysis

To test the robustness of our results, we conducted a sensitivity analysis limited to the subjects without a history of pregnancy affected by birth defects. The results did not change after removing 65 subjects with a history of pregnancy affected by birth defects (Additional file 1: Tables S5 and S6).

## Discussion

In this population-based case-control study, we examined potential association between maternal consumption of sprouted potatoes during periconceptional period and risks for NTDs and OFCs. Overall, we observed that maternal consumption of sprouted potatoes increased the risk for total NTDs, anencephaly, total OFCs, and CL ± P.

Few literatures reported the association between sprouted potato consumption and risk of NTDs in humans [24]. In our study, based on relatively large sample of NTD cases (*n* = 622) versus controls (*n* = 858), we found about 2.20-fold increased risks for NTDs among mothers who consumed sprouted potato at ≥4 meals/week and the risk increased with the rising consumption frequency of sprouted potato. One study reported that sprouted potato consumption was a risk factor affecting the occurrence of NTDs in mothers with NTD fetuses (*n* = 99) and control mothers (*n* = 99) [24]. NTD subtype analysis was absent in the previous study due to the small sample size. To our knowledge, our study is the first to examine the association between maternal sprouted potato consumption during periconceptional period and risks for NTD subtypes. We found a 2.48-fold increased risk for anencephaly not for other NTD subtypes among mothers who consumed sprouted potato at ≥4 meals/week. Several previous animal studies support our findings. Water suspensions of sprouts from seven potato varieties, gavaged at concentration of 2.5–3.5 g/kg on days 8–15 of gestation, produced NTD abnormalities in one strain of hamsters [22]. Oral administration of solanines at 0.44 mmol/kg dose on days 8–15 of gestation could induce NTDs in Syrian hamsters [23]. Glycoalkaloids were also found to induce anencephaly, microcephaly, and severe muscular kinking at a dosage of 4–5 mg/L in frog embryo [19, 20].

No previous studies have examined the role of maternal consumption of sprouted potatoes during periconceptional period in OFCs in humans. In our study, we found a 3.49-fold elevated risk for OFCs among mothers who consumed sprouted potato at ≥4 meals/week. In animal experiment, potato sprouts has been shown to be teratogenic for OFCs. Hamsters gavaged sprouts of potato suspended in water at about 2.2 ± 0.3 g/kg dose on days 8–15 of gestation presented OFC phenotypes [21].

B vitamin consumption (folic acid supplementation) has been shown a beneficial factor against the occurrence of NTDs and OFCs [26, 27]. We, therefore, examined the association between maternal sprouted potato consumption and risks of NTDs and OFCs in stratified by consumption of B vitamin. Among mother with lower consumption of B vitamins, we still found maternal consumption of sprouted potatoes significantly associated with NTDs and OFCs, however, no significant associations were observed among mother with a higher consumption of B vitamins, which meaning that B vitamins maybe antagonize the toxicity of solanine. Previous study also showed that folic acid could decrease glycoalkaloids-induced polarization, and then decrease glycoalkaloid toxicity [19].

Potato is an important food crop and constitutes an integral part of diets in many countries around the world, including China [28]. During the storage process, potato is easy to sprout in inappropriate environment and produce a large amount of solanine, which can be increased to 500 mg/100 g and is far more than the safety standard (20 mg/100 g) [29]. Solanine is stable during cooking even in frying oil at 180 °C [19]. Home processing methods (boiling, cooking, frying, and microwaving) have small and variable effects on solanine [30, 31]. Therefore, most of solanines (potato glycoalkaloids) are taken into the bodies when pregnant women have sprouted or green potatoes. The mechanisms underlying the teratogenicity of solanines are little investigated. It has been suggested that potato glycoalkaloids interfere with transport across cell membranes of Ca^2+^ and Na^+^ ions [32–35]. The potential teratogenicity of sprouted potatoes may be involved in solanine direct or indirect adverse effects on transport across cell membranes [19].

There is strength in our study. It was a relatively large population-based study with collection of the information on sprouted potato consumption and other key confounding factors, which enables us to investigate the relationship between sprouted potato consumption and NTDs with adjustment of potential interference factors. However, our study has several limitations. An intrinsic limitation of the case-control study is recall bias. Case mothers may over-report factors they believe to be risk factors of birth defects in comparison with control mothers. For assessment of recall bias, we determined whether a difference existed in the distribution of maternal consumption of sprouted potatoes during periconceptional period between women have infant with and without limb malformations. The result showed that the distribution of sprouted potato consumption between limb malformation cases and controls is similar, suggesting that recall bias of over-report was small in this study. On the other hand, to further minimize the recall bias, participants were interviewed within the first week after delivery or pregnancy termination, and the researchers helped women to confirm the period of exposure according to the dates of their last menstrual period. Second, the sample size for some subgroups was small, which limited our ability to detect statistical differences. Third, the exposure information in this study was obtained from a questionnaire. We did not measure the solanine quantity in sprouted potato. So, we can not provide data on the amount of solanine contained in a sprouted potato and the amount of solanine consumed per meal. Finally, information about consumption of dietary we collected does not specify the frequency of certain food items consumption or the weight of food consumption, and just were roughly classified by consumption of meat or fish, egg or milk, fresh vegetable, fresh fruit, and legumes in present study. So we did not use a nutrient based adjustment procedures in this study.

## Conclusions

In conclusion, maternal consumption of sprouted potatoes during periconceptional period may increase the risks for NTDs and OFCs. Our study is the first to examine the association between maternal periconceptional sprouted potato consumption and risks of NTD subtypes and OFCs in human. Our findings provide novel insight into the etiology of NTDs and OFCs and propose a new way to prevent from birth defects. Potato is a commonly consumed food items in the world, and improper preservation and use should be a matter of concern. The dietary guidance for pregnant women should be emphasized and the knowledge level of healthy fertility in reproductive woman should be improved. Maternal consumption of sprouted potato could be avoided through health education and pre-pregnancy care. Further studies aiming to replicate the findings in other racial groups and to construe the fundamental mechanism underlying the association of sprouted potato consumption and birth defects are needed.

## Additional file

Additional file 1: **Table S1.** Dietary characteristics of women who had pregnancies affected by NTDs or OFCs (cases) and women who delivered healthy infants (controls). **Table S2.** Sprouted potato consumption and risks of total NTDs and NTD subtypes stratified by consumption of B vitamin. **Table S3.** Sprouted potato consumption and risks of total OFCs and OFC subtypes stratified by consumption of B vitamin. **Table S4.** The distribution of sprouted potato consumption between Limb malformation cases and controls. **Table S5.** Sensitivity analysis: Sprouted potato consumption and risks of total NTDs and NTD subtypes. **Table S6.** Sensitivity analysis: Sprouted potato consumption and risks of total OFCs and OFC subtypes. (DOCX 34 kb)

## Abbreviations

BMI: Body mass index
CI: Confidences interval
CL ± P: Cleft lip with or without cleft palate
NTDs: Neural tube defects
OFCs: Orofacial clefts
OR: Odds radio
