# Supplementary material for: Maternal periconceptional consumption of sprouted potato and risks of neural tube defects and orofacial clefts
Source: Nutr J. 2018 Nov 28;17:112. doi: 10.1186/s12937-018-0420-4 (PMC6262956; doi:10.1186/s12937-018-0420-4)
Supplement: Supplementary file 1 — Table S1. Dietary characteristics of women who had pregnancies affected by NTDs or OFCs (cases) and women who delivered healthy infants (controls). Table S2. Sprouted potato consumption and risks of total NTDs and NTD subtypes stratified by consumption of B vitamin. Table S3. Sprouted potato consumption and risks of total OFCs and OFC subtypes stratified by consumption of B vitamin. Table S4. The distribution of sprouted potato consumption between Limb malformation cases and controls. Table S5. Sensitivity analysis: Sprouted potato consumption and risks of total NTDs and NTD subtypes. Table S6. Sensitivity analysis: Sprouted potato consumption and risks of total OFCs and OFC subtypes. (DOCX 34 kb) [file 12937_2018_420_MOESM1_ESM.docx]

**Additional file 1**

**Maternal periconceptional consumption of sprouted potato and risks of neural tube defects and** **orofacial clefts**

Wenli Ni^1^, Tian Tian^1^, Le Zhang^1^, Zhiwen Li^1^, Linlin Wang^1^*, Aiguo Ren^1^

^1^ Institute of Reproductive and Child Health, Key Laboratory of Reproductive Health, National Health Commission of the People’s Republic of China, Department of Epidemiology and Biostatistics, School of Public Health, Peking University Health Science Center, Beijing, China

^*^Correspondence to Prof. Linlin Wang, Institute of Reproductive and Child Health, Peking University, Beijing 100191, China. Telephone: +86(10)82802976. Fax: +86(10)82801141. E-mail address: [linlinwang@bjmu.edu.cn](mailto:linlinwang@bjmu.edu.cn)

Wenli Ni, E-mail address: [niwenli@bjmu.edu.cn](mailto:niwenli@bjmu.edu.cn)

Tian Tian, E-mail address: nianziruozi@163.com

Le Zhang, E-mail address: [zhangle@bjmu.edu.cn](mailto:zhangle@bjmu.edu.cn)

Zhiwen Li, E-mail address: [lizw@bjmu.edu.cn](mailto:lizw@bjmu.edu.cn)

Aiguo Ren, E-mail address: [renag@bjmu.edu.cn](mailto:renag@bjmu.edu.cn)

## Table S1 Dietary characteristics of women who had pregnancies affected by NTDs or OFCs (cases) and women who delivered healthy infants (controls)

| Characteristics | Controls (n=858) | Cases | | | |
| --- | --- | --- | --- | --- | --- |
|  |  | NTDs (n=622) | | OFCs (n=135) | |
|  | n(%)^a^ | n(%)^a^ | *P-*value | n(%)^a^ | *P-*value |
| Meat or fish |  |  |  |  |  |
| <1 meal/week | 358(41.8) | 389(62.9) | <0.001 | 69(51.5) | 0.138 |
| 1-3 meals/week | 346(40.4) | 174(28.2) |  | 46(34.3) |  |
| 4-6 meals/week | 121(14.1) | 38(6.1) |  | 13(9.7) |  |
| >6 meals/week | 31(3.6) | 17(2.8) |  | 6(4.5) |  |
| Egg or milk |  |  |  |  |  |
| <1 meal/week | 171(20.0) | 229(37.2) | <0.001 | 30(22.2) | 0.558 |
| 1-3 meals/week | 324(37.9) | 196(31.9) |  | 51(37.8) |  |
| 4-6 meals/week | 220(25.8) | 103(16.7) |  | 28(20.7) |  |
| >6 meals/week | 139(16.3) | 87(14.1) |  | 26(19.3) |  |
| Fresh vegetables |  |  |  |  |  |
| <1 meal/week | 51(6.0) | 58(9.4) | 0.028 | 6(4.5) | 0.810 |
| 1-3 meals/week | 179(21.0) | 147(23.8) |  | 26(19.5) |  |
| 4-6 meals/week | 258(30.2) | 175(28.4) |  | 39(29.3) |  |
| >6 meals/week | 365(42.8) | 237(38.4) |  | 62(46.6) |  |
| Fresh fruit |  |  |  |  |  |
| <1 meal/week | 76(8.9) | 86(13.8) | <0.001 | 12(8.9) | 0.360 |
| 1-3 meals/week | 235(27.5) | 200(32.2) |  | 28(20.7) |  |
| 4-6 meals/week | 253(29.6) | 138(22.2) |  | 41(30.4) |  |
| >6 meals/week | 291(34.0) | 197(31.7) |  | 54(40.0) |  |
| Beans or bean products |  |  |  |  |  |
| <1 meal/week | 231(27.2) | 238(38.5) | <0.001 | 38(28.4) | 0.835 |
| 1-3 meals/week | 350(41.2) | 229(37.1) |  | 52(38.8) |  |
| 4-6 meals/week | 157(18.5) | 94(15.2) |  | 23(17.2) |  |
| >6 meals/week | 112(13.2) | 57(9.2) |  | 21(15.7) |  |
|  |  |  |  |  |  |

^a^ Values for some variables may not equal the total number of cases or controls because of missing data

**Table S2** Sprouted potato consumption and risks of total NTDs and NTD subtypes **stratified by consumption of B vitamin**

| Sprouted potato consumption | Controls | Total NTDs | | | Anencephaly | | | Spina bifida | | | Encephalocele | | |
| --- | --- | --- | --- | --- | --- | --- | --- | --- | --- | --- | --- | --- | --- |
|  | n(%) | n(%) | cOR (95% CI) | aOR^a^ (95% CI) | n(%) | cOR (95% CI) | aOR^a^ (95% CI) | n(%) | cOR (95% CI) | aOR^a^ (95% CI) | n(%) | cOR (95% CI) | aOR^a^ (95% CI) |
| Lower B vitamins  consumption | | |  |  |  |  |  |  |  |  |  |  |  |
| <1 meal/week | 256 (77.6) | 242 (48.6) | 1.00 | 1.00 | 104 (75.9) | 1.00 | 1.00 | 111 (78.7) | 1.00 | 1.00 | 27 (84.4) | 1.00 | 1.00 |
| 1-3 meals/week | 64 (19.4) | 49 (15.8) | 0.81 (0.54-1.22) | 0.74 (0.45-1.21) | 22 (16.1) | 0.85 (0.50-1.45) | 0.78 (0.41-1.49) | 23 (16.3) | 0.83 (0.49-1.40) | 0.70 (0.37-1.31) | 4 (12.5) | 0.59 (0.20-1.75) | 0.78 (0.24-2.56) |
| ≥4 meals/week | 10 (3.0) | 19 (6.1) | 2.01 (0.92-4.41) | 1.84 (1.02-7.86) | 11 (8.0) | 2.71 (1.12-6.57) | 3.21 (1.00-10.35) | 7 (5.0) | 1.61 (0.60-4.35) | 2.91 (0.85-10.02) | 1 (3.1) | 0.95 (0.12-7.69) | 2.12 (0.19-23.32) |
| Higher B vitamins  consumption | |  |  |  |  |  |  |  |  |  |  |  |  |
| <1 meal/week | 481 (91.1) | 270 (86.0) | 1.00 | 1.00 | 125 (86.8) | 1.00 | 1.00 | 133 (86.9) | 1.00 | 1.00 | 11 (68.8) | 1.00 | 1.00 |
| 1-3 meals/week | 35 (6.6) | 30 (9.6) | 1.53 (0.92-2.54) | 1.54 (0.86-2.78) | 12 (8.3) | 1.32 (0.67-2.62) | 1.23 (0.53-2.84) | 14 (9.2) | 1.45 (0.76-2.77) | 1.99 (0.96-4.11) | 4 (25.0) | 4.00 (1.51-16.50) | 18.30 (0.81-415.67) |
| ≥4 meals/week | 12 (2.3) | 14 (4.5) | 2.08 (0.95-4.46) | 1.61 (0.66-3.98) | 7 (4.9) | 2.25 (0.87-5.82) | 1.39 (0.42-4.54) | 6 (3.9) | 1.81 (0.67-4.91) | 2.29 (0.76-6.86) | 1 (6.3) | 3.64 (0.44-30.53) | 5.95 (0.10-350.82) |

^a^ Adjusted for maternal age, education, BMI, occupation, infant sex, parity, season of conception, alcohol drinking, maternal smoking exposure, consumption of meat or fish, consumption of egg or milk, and consumption of legumes

## Table S3 Sprouted potato consumption and risks of total OFCs and OFC subtypes stratified by consumption of B vitamin

| Sprouted potato consumption | Controls | Total OFCs | | | CL±P | | |
| --- | --- | --- | --- | --- | --- | --- | --- |
|  | n(%) | n(%) | cOR (95% CI) | aOR^a^ (95% CI) | n(%) | cOR (95% CI) | aOR^a^ (95% CI) |
| Lower B vitamins consumption |  |  |  |  |  |  |  |
| <1 meal/week | 256(77.6) | 31(67.4) | 1.00 | 1.00 | 28(66.7) | 1.00 | 1.00 |
| 1-3 meals/week | 64(19.4) | 10(21.7) | 1.29(0.60-2.77) | 1.22(0.49-3.08) | 9(21.4) | 1.29(0.58-2.86) | 1.14(0.42-3.09) |
| ≥4 meals/week | 10(3.0) | 5(10.9) | 4.13(1.33-12.86) | 12.17(2.22-66.63) | 5(11.9) | 4.57(1.46-14.32) | 15.65(2.68-91.40) |
| Higher B vitamins consumption |  |  |  |  |  |  |  |
| <1 meal/week | 481(91.1) | 80(89.9) | 1.00 | 1.00 | 76(90.5) | 1.00 | 1.00 |
| 1-3 meals/week | 35(6.6) | 6(6.7) | 1.03(0.42-2.53) | 0.81(0.26-2.49) | 5(6.0) | 0.90(0.34-2.38) | 1.06(0.34-3.34) |
| ≥4 meals/week | 12(2.3) | 3(3.4) | 1.50(0.42-5.45) | 1.94(0.49-7.67) | 3(3.6) | 1.58(0.44-5.74) | 2.19(0.53-9.10) |

^a^ Adjusted for maternal age, education, BMI, occupation, infant sex, parity, season of conception, alcohol drinking, maternal smoking exposure, consumption of meat or fish, consumption of egg or milk, and consumption of legumes

## Table S4 The distribution of sprouted potato consumption between Limb malformation cases and controls

| Sprouted potato consumption | Controls (n%) | Limb malformations (n%) | *p*-Value |
| --- | --- | --- | --- |
|  |  |  |  |
| <1 meal/week | 737(85.9) | 87 (82.9) | 0.066 |
| 1-3 meals/week | 99(11.5) | 11 (10.5) |  |
| ≥4 meals/week | 22(2.6) | 7 (6.7) |  |

## **Table S5 Sensitivity analysis: Sprouted potato consumption and risks of total NTDs and NTD subtypes**

| Sprouted potato consumption | Controls  (n=840) | Total NTDs  (n=582) | | Anencephaly  (n=265) | | Spina bifida  (n=273) | | Encephalocele  (n=43) | |
| --- | --- | --- | --- | --- | --- | --- | --- | --- | --- |
|  | n(%) | n(%) | OR (95% CI) | n(%) | OR (95% CI) | n(%) | OR (95% CI) | n(%) | OR (95% CI) |
| Crude OR |  |  |  |  |  |  |  |  |  |
| <1 meal/week | 720(85.7) | 478(82.1) | 1.00 | 215(81.1) | 1.00 | 228(83.5) | 1.00 | 34(79.1) | 1.00 |
| 1-3 meals/week | 98(11.7) | 74(12.7) | 1.14(0.82-1.57) | 33(12.5) | 1.13(0.74-1.72) | 33(12.1) | 1.06(0.70-1.62) | 8(18.6) | 1.73(0.78-3.84) |
| ≥4 meals/week | 22(2.6) | 30(5.2) | 2.05(1.17-3.60) | 17(6.4) | 2.59(1.35-4.96) | 12(4.4) | 1.72(0.84-3.54) | 1(1.9) | 0.96(0.13-7.35) |
| Adjusted OR_1_^a^ |  |  |  |  |  |  |  |  |  |
| <1 meal/week | 720(85.7) | 478(82.1) | 1.00 | 215(81.1) | 1.00 | 228(83.5) | 1.00 | 34(79.1) | 1.00 |
| 1-3 meals/week | 98(11.7) | 74(12.7) | 1.26(0.86-1.84) | 33(12.5) | 1.26(0.76-2.08) | 33(12.1) | 1.18(0.72-1.91) | 8(18.6) | 2.30(0.92-5.74) |
| ≥4 meals/week | 22(2.6) | 30(5.2) | 2.07(1.09-3.92) | 17(6.4) | 2.32(1.09-4.96) | 12(4.4) | 1.97(0.90-4.33) | 1(1.9) | 1.26(0.15-10.23) |
| Adjusted OR_2_^b^ |  |  |  |  |  |  |  |  |  |
| <1 meal/week | 720(85.7) | 478(82.1) | 1.00 | 215(81.1) | 1.00 | 228(83.5) | 1.00 | 34(79.1) | 1.00 |
| 1-3 meals/week | 98(11.7) | 74(12.7) | 1.10(0.75-1.63) | 33(12.5) | 1.07(0.63-1.82) | 33(12.1) | 1.18(0.71-1.93) | 8(18.6) | 2.00(0.76-5.23) |
| ≥4 meals/week | 22(2.6) | 30(5.2) | 2.11(1.07-4.18) | 17(6.4) | 2.40(1.05-5.47) | 12(4.4) | 2.14(0.94-4.83) | 1(1.9) | 1.56(0.18-12.65) |

Adjusted for maternal age, education, BMI, occupation, infant sex, parity, folic acid supplementation, season of conception, alcohol drinking, and maternal smoking exposure

^b^ Adjusted for maternal age, education, BMI, occupation, infant sex, parity, folic acid supplementation, season of conception, alcohol drinking, maternal smoking exposure, consumption of meat or fish, consumption of egg or milk, consumption of fresh vegetable, consumption of fresh fruit, and consumption of legumes

## **Table S6 Sensitivity analysis: Sprouted potato consumption and risks of total OFCs and OFC subtypes**

| Sprouted potato consumption | Controls  (n=840) | Total OFCs  (n=130) | | CL±P  (n=122) | |
| --- | --- | --- | --- | --- | --- |
|  | n(%) | n(%) | OR (95 CI%) | n(%) | OR (95 CI%) |
| Crude OR |  |  |  |  |  |
| <1 meal/week | 720(85.7) | 107(82.3) | 1.00 | 101(82.8) | 1.00 |
| 1-3 meals/week | 98(11.7) | 15(11.5) | 1.03(0.58-1.84) | 13(10.7) | 0.95(0.51-1.75) |
| ≥4 meals/week | 22(2.6) | 8(6.2) | 2.45(1.06-5.64) | 8(6.6) | 2.59(1.12-5.98) |
| Adjusted OR_1_^a^ |  |  |  |  |  |
| <1 meal/week | 720(85.7) | 107(82.3) | 1.00 | 101(82.8) | 1.00 |
| 1-3 meals/week | 98(11.7) | 15(11.5) | 1.26(0.63-2.51) | 13(10.7) | 1.22(0.60-2.50) |
| ≥4 meals/week | 22(2.6) | 8(6.2) | 2.82(1.07-7.44) | 8(6.6) | 3.12(1.16-8.34) |
| Adjusted OR_2_^b^ |  |  |  |  |  |
| <1 meal/week | 720(85.7) | 107(82.3) | 1.00 | 101(82.8) | 1.00 |
| 1-3 meals/week | 98(11.7) | 15(11.5) | 1.02(0.50-2.10) | 13(10.7) | 1.09(0.51-2.32) |
| ≥4 meals/week | 22(2.6) | 8(6.2) | 3.48(1.29-9.40) | 8(6.6) | 4.02(1.45-11.17) |

^a^ Adjusted for maternal age, education, BMI, occupation, infant sex, parity, folic acid supplementation, season of conception, alcohol drinking, and maternal smoking exposure

^b^ Adjusted for maternal age, education, BMI, occupation, infant sex, parity, folic acid supplementation, season of conception, alcohol drinking, maternal smoking exposure, consumption of meat or fish, consumption of egg or milk, consumption of fresh vegetable, consumption of fresh fruit, and consumption of legumes
